# Hydrogen mobility in the lightest reversible metal hydride, LiBeH_3_

**DOI:** 10.1038/s41598-017-16504-0

**Published:** 2017-11-24

**Authors:** Eugene Mamontov, Alexander I. Kolesnikov, Sujatha Sampath, Jeffery L. Yarger

**Affiliations:** 1 Neutron Scattering Division, Oak Ridge National Laboratory, Oak Ridge, TN 37831 USA; 2 Department of Chemistry and Biochemistry, Arizona State University, Tempe, AZ 85281 USA

## Abstract

Lithium-beryllium metal hydrides, which are structurally related to their parent compound, BeH_2_, offer the highest hydrogen storage capacity by weight among the metal hydrides (15.93 wt. % of hydrogen for LiBeH_3_). Challenging synthesis protocols have precluded conclusive determination of their crystallographic structure to date, but here we analyze directly the hydrogen hopping mechanisms in BeH_2_ and LiBeH_3_ using quasielastic neutron scattering, which is especially sensitive to single-particle dynamics of hydrogen. We find that, unlike its parent compound BeH_2_, lithium-beryllium hydride LiBeH_3_ exhibits a sharp increase in hydrogen mobility above 265 K, so dramatic that it can be viewed as melting of hydrogen sublattice. We perform comparative analysis of hydrogen jump mechanisms observed in BeH_2_ and LiBeH_3_ over a broad temperature range. As microscopic diffusivity of hydrogen is directly related to its macroscopic kinetics, a transition in LiBeH_3_ so close to ambient temperature may offer a straightforward and effective mechanism to influence hydrogen uptake and release in this very lightweight hydrogen storage compound.

## Introduction

Metal hydrides have several attractive features as hydrogen storage media, such as high volumetric storage density and high cycling capability, but improving their gravimetric storage density remains a challenge. In search for the reversible metal hydrides with hydrogen gravimetric storage density exceeding that of MgH_2_ (7.65 wt. %), one has to turn to hydrides of beryllium, since the hydride of monovalent sodium, NaH, has a lower hydrogen weight storage capacity compared to that of MgH_2_, and lithium hydride, LiH, is practically irreversible^1^. Beryllium hydride, BeH_2_ (18.28 wt. % of hydrogen), is a parent compound for a class of complex lithium-beryllium hydrides, the most promising of which, LiBeH_3_, has 15.93 wt. % of hydrogen. Despite the great promise for reversible hydrogen storage, synthesis of BeH_2_ and the derivative lithium-beryllium compounds is so cumbersome that to date their crystallographic analysis has been beset with difficulties and remains inconclusive, even though they have been and continue to be studied extensively to better understand their phase behavior^2–12^. It is believed that the parent structure of BeH_2_ consists of corner-sharing BeH_4_ tetrahedral units^13–20^ and may exhibit polymorphism, similar to various forms of silica. Likewise, LiBeH_3_ is generally believed to feature BeH_4_ tetrahedral units^21,22^, although BeH_6_ octahedral units have been also proposed to describe its structure^23^. Various crystallographic group assignments have been proposed for LiBeH_3_, as described in earlier studies^21–27^. The accurate determination of its structure is complicated by unavailability of single crystals and low x-ray scattering power of light atoms, particularly hydrogen. Neutron diffraction studies, which could potentially overcome the latter problem and accurately assess positions occupied by hydrogen atoms in the LiBeH_3_ structure, would require deuterated compounds. If they could be determined, local structural parameters, such as H-H distances, would be relevant to the microscopic hydrogen diffusion dynamics and ultimately the macroscopic kinetics of hydrogen uptake and release. This applies not only to crystalline, but also amorphous, lithium-beryllium compounds, which are easier to synthesize and might be more suitable for applications. Together with infrared and Raman spectroscopy, inelastic neutron spectroscopy was used in the past to characterize the vibrational dynamics in amorphous beryllium and lithium-beryllium hydrides^28^. However, a separate measurement would be needed to probe hydrogen diffusivity in these hydrides. Here we investigate momentum transfer dependence of quasielastic neutron scattering (QENS) signal, which is particularly sensitive to single-particle diffusion dynamics of hydrogen due to its large incoherent neutron scattering cross-section, to elucidate the spatial and temporal characteristics of hydrogen diffusion jumps in BeH_2_ and LiBeH_3_.

## Results and Discussion

**Figure 1:**
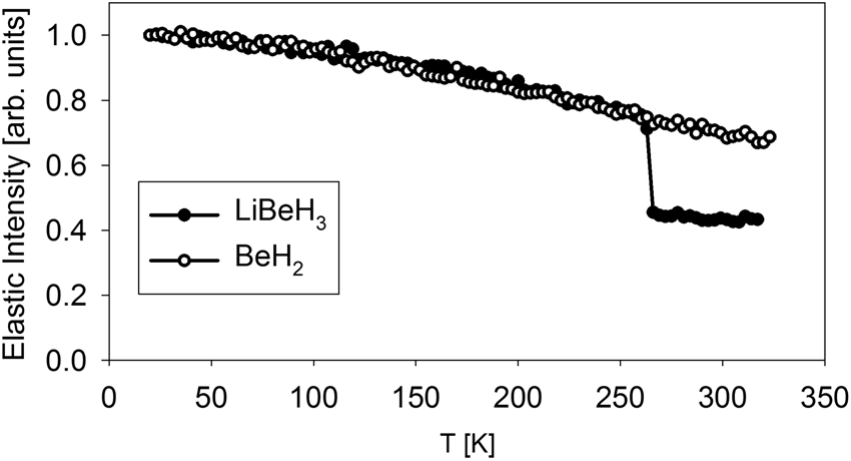
Temperature dependence of the elastically scattered (within the resolution limit of the spectrometer) neutron intensity averaged over the entire investigated momentum transfer range and normalized to unity at the baseline temperature of 20 K. The spectra, dominated by the incoherent scattering from the hydrogen in the hydrides, show a sharp melting-type transition for LiBeH_3_ at ca. 265 K.

The temperature dependence of the elastic (within the resolution of the spectrometer) neutron intensity is presented in Fig. 1. The data normalized to the lowest measured temperature overlap up to ca. 265 K, when LiBeH_3_ exhibits a dramatic drop in the elastic intensity. The elastic intensity does not drop to a near-zero value, as would be the case for a complete melting of the sample. Nevertheless, a sudden change in the state of the hydrogen sublattice, which dominates the scattering signal, is evident. The sharpness of the elastic intensity drop is remarkable; it surpasses, e.g., the already rather sharp elastic intensity drop due to the onset of rapid oxygen diffusivity in bismuth oxide above ca. 740 °C underlined by a phase transition to δ-Bi_2_O_3_ structure^29^, while most order-disorder transitions observed in ion conductors are much more gradual. The abrupt transition in the hydrogen sublattice in LiBeH_3_ is reminiscent of partial melting. To this end, it is of interest to investigate the mechanism of hydrogen hopping in BeH_2_ and LiBeH_3_ at various temperatures. Unlike the data presented in Fig. 1, which are completely model-independent, such analysis of microscopic hydrogen diffusivity has to rely on fitting QENS data with a suitable model scattering function. In the simplest case, the model scattering function in QENS is a superposition of Lorentzian terms, where each Lorentzian term in the energy transfer space is a Fourier transformation of the corresponding term in the time space describing exponential decay with time of self-correlation for diffusing particles. For a comprehensive overview of QENS technique and data analysis, see^30^.

The QENS data, I(Q, E), collected as a function of energy and momentum transfer, E and Q, are fitted with the following model scattering function:

1 $$I(Q,E)=[x(Q)\delta (E)+(1-x(Q))S(Q,E)]\otimes R(Q,E)+({C}_{1}(Q)E+{C}_{2}(Q))$$

The fits of the scattering intensity, I(Q, E), are performed separately at each Q value, and include a superposition of elastic line (delta-function) with a spectral weight x(Q) and a quasielastic model scattering function term, S(Q, E), with the complementary spectral weight 1−x(Q), convolved with the resolution function, R(Q, E), and a linear background term, (C_1_(Q)E + C_2_(Q)). The model scattering functions used in the current study included either one, or two Lorentzian terms, as follows:

2 $$S(Q,E)=p(Q)\frac{1}{\pi }\frac{HWHM{(Q)}_{narrow}}{HWH{M}^{2}{(Q)}_{narrow}+{E}^{2}}+(1-p(Q))\frac{1}{\pi }\frac{HWHM{(Q)}_{broad}}{HWH{M}^{2}{(Q)}_{broad}+{E}^{2}}$$

**Figure 2:**
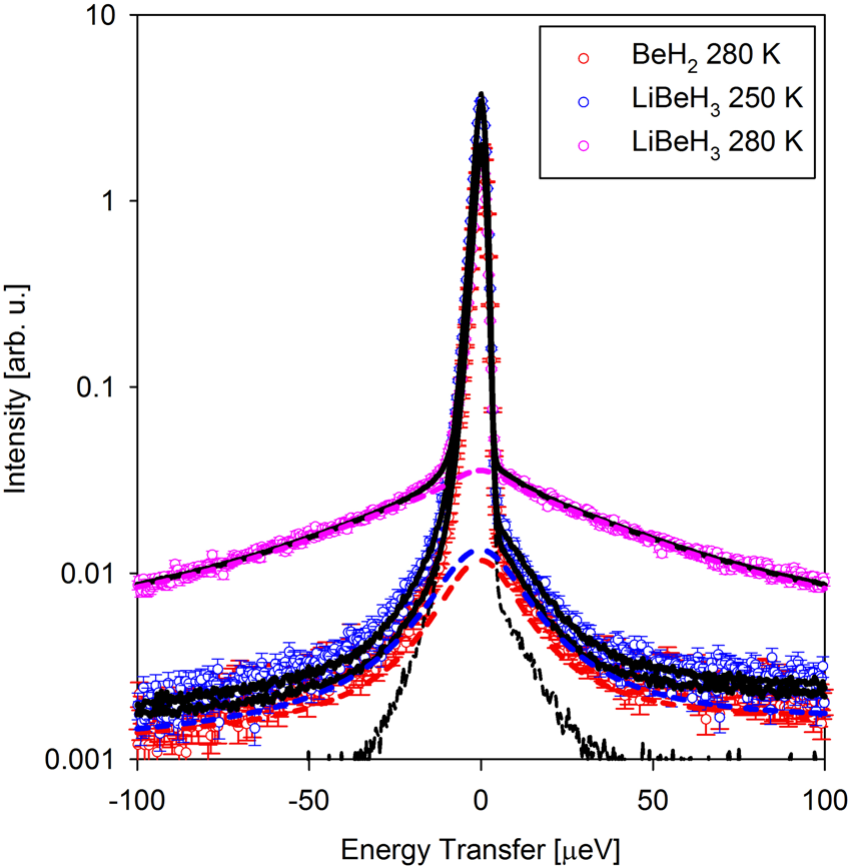
QENS spectra (symbols) and overall fits (black solid lines) obtained as described in the text at a representative Q = 0.9 Å^−1^. The inelastic components of the fits (QENS components plus backgrounds) are shown with the colored dashed lines. The resolution function is shown with a black dashed line.

**Figure 3:**
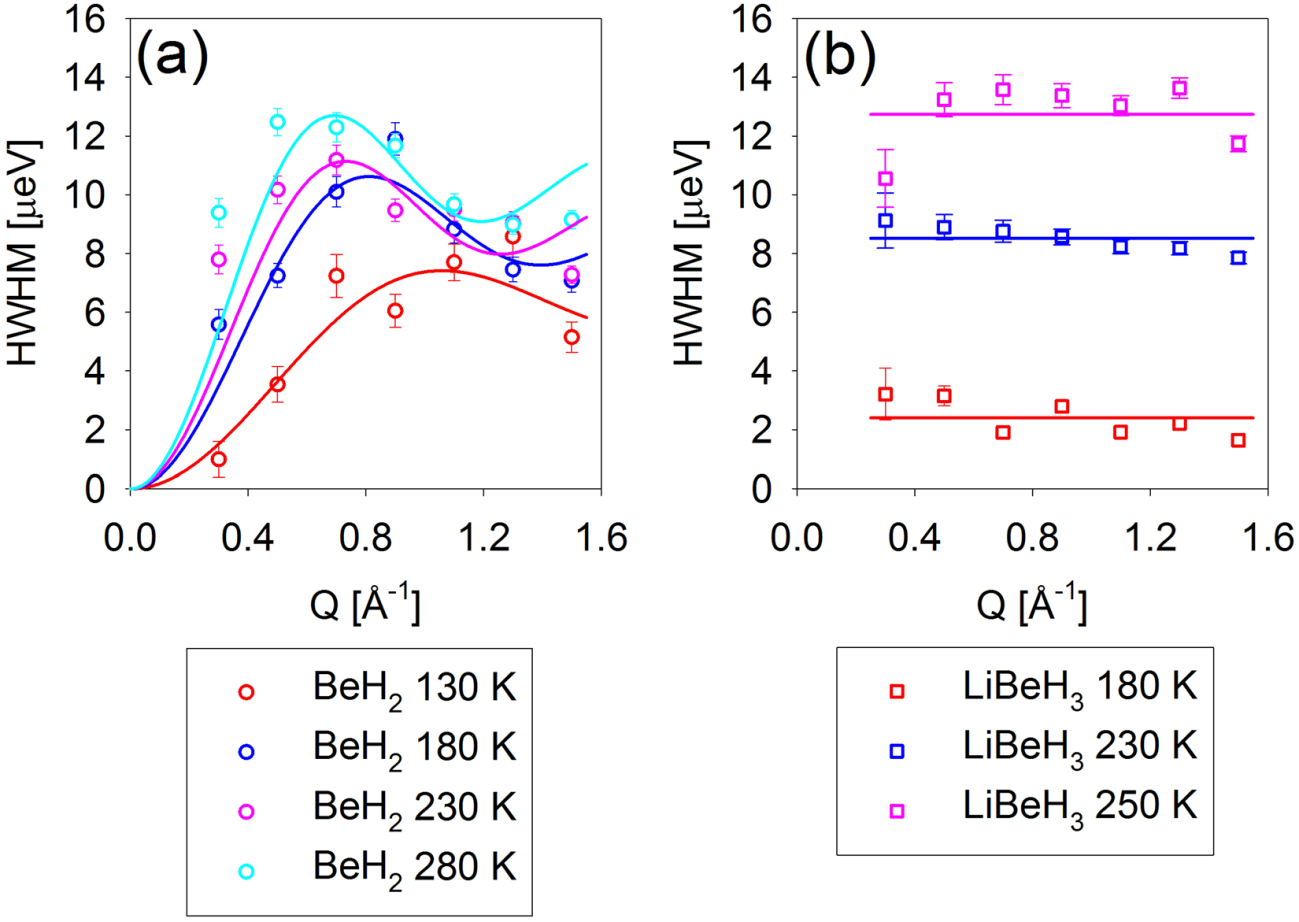
Symbols: Q-dependence of the Lorentzian term describing the QENS broadening for BeH_2_ (**a**) and LiBeH_3_ (**b**). The former exhibits a pronounced increase at low Q and a weak maximum in the mid-Q range and thus can be fitted with a jump-diffusion model (solid lines). For the latter, average values over the Q-range are taken (solid lines), since the largely Q-independent data are indicative of localized jumps.

**Figure 4:**
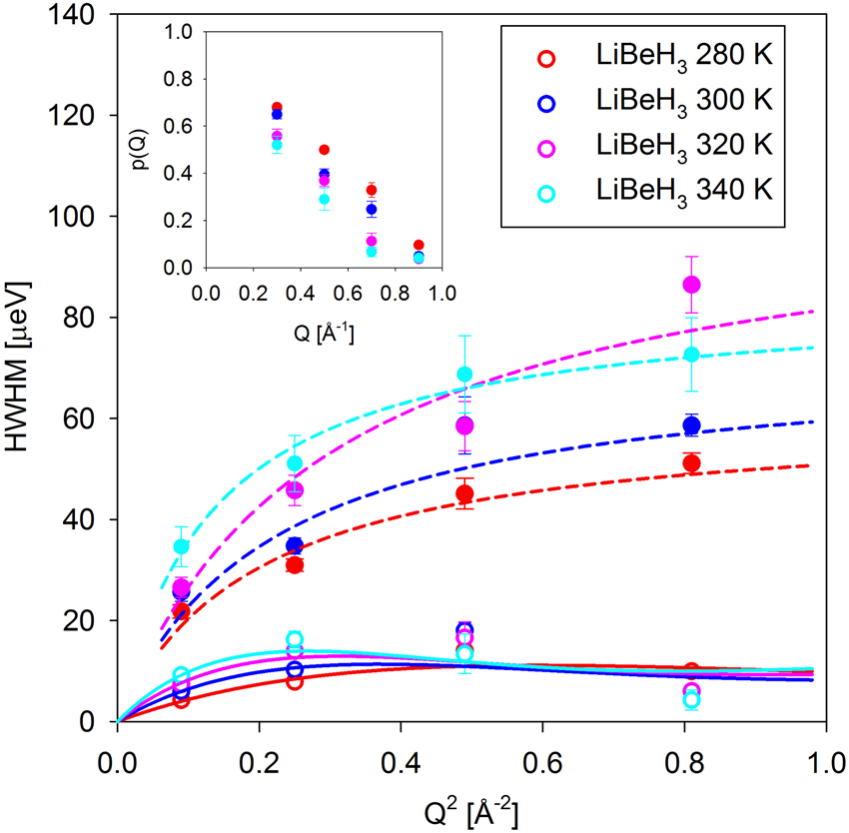
Q-dependence of the narrower and broader Lorentzian terms describing the QENS broadening observed for LiBeH_3_ above the transition. For the narrower Lorentzian, it can be fitted with a jump-diffusion model (solid lines). For the broader Lorentzian, it can be fitted with a jump-diffusion model averaged over a distribution of jump lengths, known as a liquid diffusion model (dashed lines). Inset: the relative spectral weight of the narrower Lorentzian.

The drastic difference of the scattering signal from LiBeH_3_ observed above the transition is illustrated by Fig. 2. A simple one-Lorentzian fits adequately described the data for BeH_2_ and LiBeH_3_ below the transition; the resulting Q-dependence of the Lorentzian half-width at half-maximum, HWHM(Q), is presented in Fig. 3. The data for BeH_2_ exhibit stronger Q-dependence compared to those for LiBeH_3_. In particular, they show an increase at low Q followed by a weak maximum in the mid-Q range, which suggests that the hydrogen hopping in BeH_2_ can be described by Chudley-Elliott jump diffusion model^30^ (Fig. 3a):

3 $$HWHM(Q)=\frac{6\hslash D}{{L}^{2}}(1-\frac{\sin (QL)}{QL})$$

where *ℏ* is reduced Planck constant, D is the diffusion coefficient and L is the diffusion jump length. Chudley-Elliott jump diffusion model assumes that, for some characteristic time between jumps, τ, a particle engages only in vibrational motions near an equilibrium position, and then relatively quickly (compared to the time between jumps) performs a diffusion jump into a new equilibrium position. The jump length and diffusion coefficient are related to the characteristic time between jumps, τ, as D = L^2^/6τ; they are listed in Table 1. The HWHM(Q) values for LiBeH_3_ below the transition are relatively Q-independent, indicative of localized, rather than long-range translational, hydrogen jumps. They can be described by the values averaged over the measured Q-range, <HWHM(Q)> (Fig. 3b), which can be converted to the characteristic time between jumps as τ = *ℏ*/<HWHM(Q)>. On the other hand, a two-Lorentzian fit was needed to adequately describe the data for LiBeH_3_ above the transition. The resulting HWHM(Q) of the narrower Lorentzian can still be fitted with Chudley-Elliott jump diffusion model, but the HWHM(Q) of the broader Lorentzian is different in character; while increasing with Q, it lacks a maximum in the mid-Q range (Fig. 4). Thus, it has to be described by a liquid-diffusion model^30^:

4 $$HWHM(Q)=\frac{6\hslash D}{ < {L}^{2} > }(1-\frac{1}{1+{Q}^{2} < {L}^{2} > /6})$$

where the mean squared diffusion jump length < L^2^ >  = 6Dτ. Liquid-diffusion model (Equation 4) can be derived^30^ from Chudley-Elliott jump diffusion model (Equation 3) by assuming an exponential distribution of jump lengths, L.

**Table 1:** Jump diffusion fit parameters.

T (K)	D (×10^−10^ m^2^/s)	τ (×10^−9^ s)	L (Å)
BeH_2_			
130	2.8 (0.4)	0.108 (0.004)	4.24 (0.21)
180	6.8 (0.5)	0.075 (0.002)	5.54 (0.15)
230	8.8 (0.5)	0.072 (0.001)	6.14 (0.13)
280	11.1 (0.6)	0.063 (0.001)	6.48 (0.12)
LiBeH_3_ below transition			
180		0.292 (0.081)	
230		0.082 (0.011)	
250		0.054 (0.007)	
LiBeH_3_ above transition, slow component			
280	7.9 (1.1)	0.072 (0.004)	5.84 (0.25)
300	12.9 (1.7)	0.071 (0.003)	7.39 (0.30)
320	17.3 (2.6)	0.062 (0.003)	8.01 (0.40)
340	21.8 (5.9)	0.057 (0.005)	8.67 (0.75)
LiBeH_3_ above transition, fast component			
280	46.3 (4.5)	0.011 (0.001)	5.53 (1.04)
300	50.5 (5.4)	0.009 (0.001)	5.22 (1.14)
320	54.2 (6.0)	0.006 (0.001)	4.42 (1.23)
340	94.1 (21.4)	0.008 (0.001)	6.72 (2.37)

Standard deviation values are shown in parenthesis.

Hydrogen hopping mechanism in LiBeH_3_ above the transition is highly unusual in that it exhibits characteristic traits of both solid state and liquid diffusion processes. Resemblance to the solid-state diffusion is evident from the fact that the HWHM(Q) for the narrower Lorentzian, which is typically associated with the long-range translational diffusivity, is described by a solid-state Chudley-Elliott jump diffusion model with a well-defined jump length, not a liquid-diffusion model with a distribution of jump lengths. However, resemblance to the liquid diffusion is evidenced by the strongly Q-dependent spectral weight of the narrow Lorentzian (parameter p(Q) in Equation 2), as presented in Fig. 4 inset. Such Q-dependence of the p(Q) indicates that the two observed Lorentzian components originate from the same diffusing entity^31–33^, and not two separate diffusing species; in the latter case, a Q-independent Lorentzian spectral weight would be expected. In liquids, the narrow Lorentzian is associated with the long-range translational diffusivity, whereas the broad Lorentzian is due to the localized center-of-mass motions in the transient cage made by the neighboring liquid particles^31–33^. These localized motions give rise to a broad Q-dependent component, which should, however, plateau at low Q. No leveling off is observed for the broad component presented in Fig. 4, which may indicate that the characteristic volume associated with the localized diffusivity of hydrogen in LiBeH_3_ above the transition could be too large to be detected within the Q range of our experiment.

The diffusion jump lengths for hydrogen in BeH_2_ and LiBeH_3_ above the transition (slow component) presented in Table 1 exhibit rather strong and systematic temperature dependence, consistent with long-range hydrogen diffusivity. On the other hand, the diffusion jump length associated with the localized motions of hydrogen in LiBeH_3_ above the transition (fast component) does not exhibit a pronounced temperature dependence, as expected for localized motions in the liquid state.

**Figure 5:**
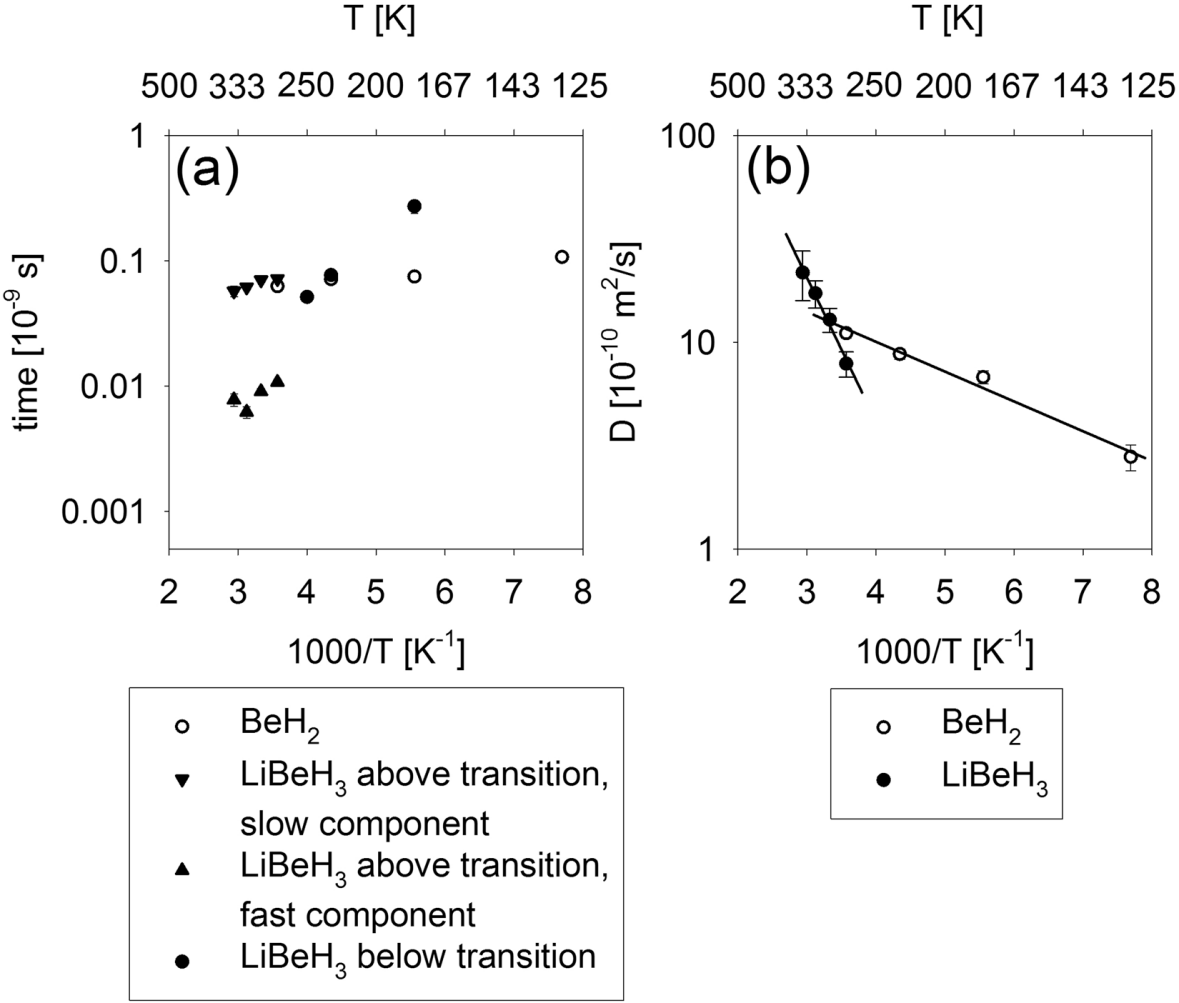
Symbols: Characteristic times between jumps (**a**) and long-range diffusion coefficients (**b**) obtained from the data fits. Solid lines are Arrhenius temperature dependence fits.

Figure 5 shows Arrhenius plots of the characteristic times between jumps and the long-range diffusion coefficients. The long-range diffusion is not measurable for the localized hydrogen hopping in LiBeH_3_ below the transition. Furthermore, here we chose not to display the diffusivity values listed in Table 1 for the fast component measured in LiBeH_3_ above the transition, because of their likely association with localized rather than long-range diffusivity. The activation energy for the long-range translational diffusivity (solid lines in Fig. 5b) is (2.8 ± 0.3) kJ/mol and (13.3 ± 1.1) kJ/mol for BeH_2_ and LiBeH_3_, respectively. Likewise, even below the transition, where hydrogen in LiBeH_3_ engages only in localized hopping, its associated activation energy of the residence times appears much larger compared to that of the residence times for hydrogen in BeH_2_, as evidenced by much steeper temperature dependence seen at low temperatures in Fig. 5a.

It is unclear as to whether the unusual character of hydrogen hopping in LiBeH_3_ above the transition is related to the amorphous structure of this hydride. On the one hand, it is tempting to attribute the unusual resemblance of hydrogen mobility in this hydride to both liquid- and solid-state diffusion to the fact that the LiBeH_3_ structure is amorphous, characterized by the local order and long-range disorder, which can introduce a variety of jump distances. On the other hand, BeH_2_, which is also amorphous, does not exhibit any sudden transition in the hydrogen mobility. If the amorphous structure of a hydride is indeed essential for the unusual hydrogen hopping mechanism as reported here, there is a possibility that other amorphous compounds derived from BeH_2_ may exhibit similar mechanism of hydrogen diffusivity, including the transition, if they happen to remain thermodynamically stable to the sufficiently high temperature.

In summary, a sharp increase in the hydrogen mobility observed in LiBeH_3_ above 265 K is associated with the abrupt transition of the hydrogen sublattice to a peculiar state that exhibits characteristic traits of both solid-like and liquid-like diffusivity. Because this transition is so close to ambient temperature, it can be used as a mechanism to regulate hydrogen diffusivity in LiBeH_3_. Ultimately, it may help control hydrogen uptake and release in this very lightweight hydrogen storage compound, even though we should note that our QENS measurements were performed in the temperature range of the sample stability, and thus were not directly associated with hydrogen uptake and release.

## Materials and Methods

### Materials

BeH_2_ and LiBeH_3_ materials investigated in the current study were the same samples as described in earlier work^28^, which refers to the materials synthesis procedures. In general, solid BeH_2_ had primarily been synthesized from decomposition of organometallic precursors^34–36^. This synthesis method typically produces amorphous products, which can be transformed to crystalline phases at elevated pressure and temperature. Pressure is required to keep the solid amorphous BeH_2_ from decomposing into its elemental constituents. Both BeH_2_ crystalline phases and the amorphous solid material have been shown to consist of network of corner-sharing BeH_4_ tetrahedral units^17^.

### QENS Data Collection and Reduction

Quasielastic neutron scattering (QENS) experiment was carried out using high energy resolution backscattering spectrometer BASIS^37^ at the Spallation Neutron Source (SNS) at Oak Ridge National Laboratory. The energy resolution was 3.4 μeV (full width at half-maximum) for the Q-averaged resolution value, and the energy transfer window suitable for the data analysis was ±100 μeV. The minimum and maximum momentum transfer, Q, value suitable for data analysis was 0.3 Å^−1^ and 1.5 Å^−1^, respectively. The powder samples contained in aluminum foil were placed in annular aluminum sample holders. Initially, each sample was cooled down to the baseline temperature of 20 K to measure the spectra (for several hours each) used for the sample-specific resolution function under the conditions when the measurable hydrogen hopping had ceased. Then the temperature was ramped up at a rate of 1 K/min while the data almost continuously collected for about 5 hours were integrated over ±3.4 μeV energy transfer range to obtain the temperature dependence of the elastically scattered neutron intensity (averaged over all Q values). Then longer dynamic measurements (several hours each) were performed at a few temperatures chosen to remain below the known limits of thermal decomposition^38^. A vanadium standard was used for data normalization.

### Data Availability

The datasets analyzed during the current study are available from the corresponding author on reasonable request.

## References

[1] Weast, R. C. (Ed.), Handbook of Chemistry and Physics, CRC Press, OH, USA (1975).

[2] Ahart M (2006). High-pressure Brillouin scattering of amorphous BH_2_. J. Chem. Phys..

[3] Harder S (2012). Molecular early main group metal hydrides: synthetic challenge, structures and applications. Chem. Commun..

[4] Chen Y (2013). Exploring High-Pressure Lithium Beryllium Hydrides: A New Chemical Perspective. J. Phys. Chem. C.

[5] Wang Z (2014). Metallization and superconductivity of BeH_2_ under high pressure. J. Chem. Phys..

[6] Zdetsis AD, Sigalas MM, Koukaras EN (2014). *Ab initio* theoretical investigation of beryllium and beryllium hydride nanoparticles and nanocrystals with implications for the corresponding infinite systems. Phys. Chem. Chem. Phys..

[7] Pardanaud C (2015). Hydrogen retention in beryllium: concentration effect and nanocrystalline growth. J. Phys.: Condens. Matter.

[8] Koukaras EN, Sgouros AP, Sigalas MM (2016). Fully Hydrogenated Beryllium Nanoclusters. J. Am. Chem. Soc..

[9] Guendouz D (2016). Electronic structure, optical and thermodynamic properties of ternary hydrides MBeH_3_ (M = Li, Na, and K). Can. J. Phys..

[10] Pepin CM, Loubeyre P (2016). Layered structure and re-entrant disproportionation observed in crystalline BeH_2_ under pressure. Phys. Rev. B.

[11] Rehmat B (2017). Elastic properties of perovskite-type hydrides LiBeH_3_ and NaBeH_3_ for hydrogen storage. Int. J. Hydrogen Energy.

[12] An X, Zeng T, Ren W (2017). Structural, electronic and optical properties of BeH2: A density functional theory study. Mater. Res. Express.

[13] Marchenko SV, Petrunin VF, Markushkin YE, Senin MD, Chirin NA (1982). Neutron scattering study of amorphous beryllium hydride. Fizika Tverdogo Tela.

[14] Smith GS (1988). The crystal and molecular structure of beryllium hydride. Solid State Commun..

[15] Yu R, Lam PK, Head J (1989). Lattice dynamics of crystalline beryllium hydride. Solid State Commun..

[16] Senin MD (1993). The production, structure, and properties of beryllium hydride. Inorg. Mater..

[17] Sampath S (2003). Structural quantum isotope effects in amorphous beryllium hydride. J. Chem. Phys..

[18] Hector LG, Herbst JF, Wolf W, Saxe P, Kresse G (2007). *Ab Initio* thermodynamic and elastic properties of alkaline-earth metals and their hydrides. Phys. Rev. B.

[19] Bachurin DV, Vladimirov PV (2016). Simulation of hydrogen effect on equilibrium shape of gas bubbles in beryllium. Fusion Eng. Des..

[20] Virot, F., Ferry, L., Ferro, Y., Pardanaud, C. & Barrachin, M. Contribution to a better evaluation of the dust speciation in case of an accident in ITER. *Fusion Eng. Des*., in press, 10.1016/j.fusengdes.2017.02.102 (2017).

[21] Martins JL (1988). Electronic and structural properties of LiBeH_3_. Phys. Rev. B.

[22] Hu C-H (2008). Crystal structure prediction of LiBeH_3_ using *ab initio* total-energy calculations and evolutionary simulations. J. Chem. Phys..

[23] Vajeeston P, Ravindran P, Fjellvag H (2008). Structural Phase Stability Studies on MBeH_3_ (M = Li,Na,K,Rb,Cs) from Density Functional Calculations. Inorg. Chem..

[24] Overhauser AW (1987). Crystal structure of lithium beryllium hydride. Phys. Rev. B.

[25] Selvam P, Yvon K (1989). Comment on “Crystal structure of lithium beryllium hydride”. Phys. Rev. B.

[26] Cantrell JS, Beiter TA, Souers PC, Barry P (1991). Crystallographic studies of compounds from the LiH + BeH_2_ system. J. Less-Comm. Metals.

[27] Zaluska A, Zaluski L, Strom-Olsen JO (2000). Lithium-beryllium hydrides: the lightest reversible metal hydrides. J. Alloys Cmpnds..

[28] Sampath S, Kolesnikov AI, Lantzky KM, Yarger JL (2008). Vibrational dynamics of amorphous beryllium hydride and lithium beryllium hydrides. J. Chem. Phys..

[29] Mamontov E (2016). Fast oxygen diffusion in bismuth oxide probed by quasielastic neutron scattering. Solid State Ionics.

[30] Bee, M. Quasielastic Neutron Scattering, Hilger, Bristol (1988).

[31] Mamontov E, Luo H, Dai S (2009). Proton Dynamics in *N,N,N’,N’*-Tetramethyl-Guanidinium Bis(perfluoroethylsulfonyl)imide Protic Ionic Liquid Probed by Quasielastic Neutron Scattering. J. Phys. Chem. B.

[32] Qvist J, Schober H, Halle B (2011). Structural Dynamics of Supercooled Water from Quasielastic Neutron Scattering and Molecular Simulations. J. Chem. Phys..

[33] Mamontov E (2012). Diffusion in confinement as a microscopic relaxation mechanism in glass-forming liquids. Chem. Phys. Lett..

[34] Coates, G. E. & Glocking, F. Diisopropylberyllium and Some Beryllium Hydrides. *J. Chem. Soc*., 22–27, 10.1039/JR9540000022 (1954).

[35] Coates, G. E. & Glocking, F. Di-tert. -butylberyllium and Beryllium Hydride. *J. Chem. Soc*., 2526–2529, 10.1039/JR9540002526 (1954).

[36] Baker RW (1978). Preparation of Beryllium Hydride by an Improved Pyrolysis Technique. J. Organomet. Chem..

[37] Mamontov E, Herwig KW (2011). A Time-of-Flight Backscattering Spectrometer at the Spallation Neutron Source, BASIS. Rev. Sci. Inst..

[38] Grochala W, Edwards PP (2004). Thermal Decomposition of the Non-Interstitial Hydrides for the Storage and Production of Hydrogen. Chem. Rev..

